# A decade of progress in type 2 diabetes and cardiovascular disease: advances in SGLT2 inhibitors and GLP-1 receptor agonists – a comprehensive review

**DOI:** 10.3389/fendo.2025.1605746

**Published:** 2025-07-07

**Authors:** David Aristizábal-Colorado, David Corredor-Rengifo, Santiago Sierra-Castillo, Carolina López-Corredor, David-Alexander Vernaza-Trujillo, Danilo Weir-Restrepo, Juan S. Izquierdo-Condoy, Esteban Ortiz-Prado, Jorge Rico-Fontalvo, Juan-Esteban Gómez-Mesa, Alin Abreu-Lomba, Wilfredo-Antonio Rivera-Martínez

**Affiliations:** ^1^ Internal Medicine Department, Universidad Libre, Cali, Colombia; ^2^ Grupo Interinstitucional de Medicina Interna (GIMI1), Universidad Libre, Cali, Colombia; ^3^ Epidemiology Department, Universidad Centros de Estudios en Salud (CES), Medellín, Colombia; ^4^ Medicine Program, Universidad Santiago de Cali, Cali, Colombia; ^5^ Department of Public Health, Pontificia Universidad Javeriana, Cali, Colombia; ^6^ Clinical Research Center, Clínica Imbanaco, Cali, Colombia; ^7^ Internal Medicine Department, Universidad Centros de Estudios en Salud (CES), Medellin, Colombia; ^8^ One Health Research Group, Universidad de las Américas, Quito, Ecuador; ^9^ Department of Nephrology, Faculty of Medicine, Universidad Simón Bolívar, Barranquilla, Colombia; ^10^ Latin American Society of Nephrology and Arterial Hypertension (SLANH), Ciudad de Panama, Panama; ^11^ Cardiology Department, Fundación Valle del Lili, Cali, Colombia; ^12^ Department of Health Sciences, Universidad Icesi, Cali, Colombia; ^13^ Endocrinology Department, Clínica Imbanaco, Cali, Colombia; ^14^ Endocrinology Department, Universidad de Antioquia, Medellín, Colombia

**Keywords:** cardiovascular outcomes, SGLT2 inhibitors, GLP-1 agonists, combination therapy, heart failure, renal outcomes

## Abstract

Cardiovascular and renal complications remain leading causes of morbidity and mortality among individuals with type 2 diabetes mellitus (T2DM). Since 2015, large-scale cardiovascular outcome trials (CVOTs) have demonstrated that sodium-glucose cotransporter-2 inhibitors (SGLT2i) and glucagon-like peptide-1 receptor agonists (GLP-1 RAs) significantly reduce the risk of major adverse cardiovascular events, cardiovascular mortality, and heart failure hospitalization in patients with T2DM and established cardiovascular disease or high-risk profiles. These findings—originating from landmark trials such as EMPA-REG OUTCOME, LEADER, and SUSTAIN-6—have led to substantial revisions in international guidelines from the European Society of Cardiology, American College of Cardiology, and American Heart Association, which now recommend the use of SGLT2i or GLP-1 RAs, often in conjunction with metformin. SGLT2i have shown robust effects in reducing heart failure hospitalization and slowing the progression of chronic kidney disease, while GLP-1 RAs have demonstrated superior efficacy in reducing atherothrombotic events, particularly non-fatal stroke. Additionally, emerging data supports the complementary use of both drug classes, revealing additive benefits on cardiovascular and renal outcomes without increased toxicity. This narrative review summarizes the mechanisms of action, clinical efficacy, safety profiles, and sex-specific outcomes associated with SGLT2i and GLP-1 RAs. It also highlights key evidence supporting their combined use and underscores their critical role in optimizing long-term outcomes in patients with T2DM and cardiovascular disease.

## 1 Introduction

Cardiovascular disease (CVD) continues to be a significant global health concern, with ischemic stroke and acute myocardial infarction ranking as the second and third leading causes of death worldwide in 2019 (1). In 2020, an estimated 523 million people were affected by CVD, resulting in approximately 19 million deaths—an 18.7% increase compared to 2010 (2). In parallel, type 2 diabetes mellitus (T2DM) continues to rise, with 536.6 million individuals affected globally in 2021. This figure is projected to increase to 783.2 million by 2045 (3). The CALIBER UK study highlights that peripheral arterial disease (16.2%), heart failure, stable angina, non-fatal myocardial infarction, and cerebrovascular accidents are common cardiovascular complications in T2DM patients after 5.5 years of follow-up (4).

The complex interaction between diabetes mellitus (DM) and cardiovascular events complicates patient management. Individuals with T2DM frequently have multiple cardiovascular risk factors, including obesity (32.9%), hypertension (32-80%), and dyslipidemia (39%) (5–8). Moreover, factors such as oxidative stress, hypercoagulability, endothelial dysfunction, and autonomic neuropathy contribute significantly to CVD risk in T2DM patients (5).

The association between glycemic control and cardiovascular outcomes in DM patients has been long studied (9–11). However, recent evidence shows conflicting results regarding the benefits of strict glycemic control on reducing cardiovascular events. Although stringent glycemic targets (HbA1c ≤ 6.5%) may yield benefits on microvascular complications, their impact on cardiovascular mortality remains uncertain (12, 13). Moreover, pursuing overly strict control may increase the risk of hypoglycemia, weight gain, and all-cause mortality (14, 15).

To address these challenges, novel pharmacological therapies such as sodium-glucose cotransporter-2 inhibitors (SGLT-2i) and glucagon-like peptide-1 receptor agonists (GLP-1 RAs) have emerged, offering cardioprotective benefits (16, 17). These agents have been shown to reduce cardiovascular mortality, slow chronic kidney disease (CKD) progression, and decrease heart failure (HF) hospitalizations (17–19). Leading clinical guidelines now recommend the combined or monotherapy use of SGLT-2i and GLP-1 RAs in patients with T2DM and CVD or those at high cardiovascular risk (20, 21).

The aim of this review is to enhance our understanding of the role of SGLT-2i and GLP-1 RAs in managing patients with T2DM and CVD. By highlighting key findings and clinical implications, this review aims to provide valuable information for healthcare professionals involved in the care of these patients.

## 2 Material and methods

This narrative literature review examines the use of SGLT-2 inhibitors and GLP-1 RAs in patients with T2DM and CVD. The review included a comprehensive search of peer-reviewed articles published between January 2010 and November 2024 conducted using the PubMed and Medline databases, supplemented by the inclusion of relevant studies from earlier periods when clinically justified. Articles were primarily selected based on clinical relevance and their inclusion in current international guidelines for the management of patients with CVD and T2DM. The search strategy incorporated the following Boolean logic; (SGLT-2 inhibitors) AND (GLP-1 receptor agonists) AND (type 2 diabetes mellitus) AND (cardiovascular diseases).

The review process comprised three distinct stages:

1. An exhaustive search of documentary material using PubMed and Medline databases.
2. Classification and selection of the most relevant articles based on predefined evaluation criteria.
3. Detailed analysis and synthesis of the extracted data.

The initial search yielded 564 articles. Inclusion criteria were studies involving adult patients (≥18 years) with T2DM and CVD; evaluation of SGLT-2 inhibitors or GLP-1 receptor agonists; and reporting of cardiovascular or renal outcomes. Eligible study designs included randomized controlled trials, observational studies (cohort or case-control), systematic reviews, and meta-analyses. Only peer-reviewed, indexed publications were considered. Data extraction and review were independently conducted by three authors (DAC, DCR, and SSC) using a standardized data collection form. A total of 407 records were excluded based on title and abstract screening, and 113 full-text articles were assessed for eligibility. The final manuscript was reviewed by experts in endocrinology, nephrology, and cardiology, who provided critical feedback and ensured comprehensive bibliographic coverage.

## 3 Results

### 3.1 Mechanism of action and clinical impact

#### 3.1.1 Mechanism of action of SGLT2 inhibitors

Phlorizin, a botanical extract, is a non-specific inhibitor of sodium-glucose transporter proteins. Its discovery traces back over 150 years to research on glucosuria (22). Since then, various types of SGLT proteins have been identified. SGLT2i, in particular, target the sodium-glucose cotransporter 2, which is primarily located in the proximal tubular epithelium. This protein is responsible for roughly 90% of renal glucose reabsorption, and by inhibiting it, SGLT2i effectively disrupt this reabsorption process (23).

By blocking sodium-glucose cotransporter 2 in the S1 and S2 segments of the proximal tubule, SGLT2i significantly reduce glucose reabsorption, promoting urinary glucose excretion. This creates a state of “relative hypoglycemia,” which has several beneficial effects, including reductions in both systolic and diastolic blood pressure through decreased circulating volume and improved glomerular hyperfiltration control. Additionally, SGLT2i can lower HbA1c levels by 0.5-1.0% and support weight loss (24, 25). Increased natriuresis and sodium delivery to the distal nephron, induced by SGLT2 inhibition, are key in renal protection, normalizing the tubuloglomerular feedback mechanism, a principal driver of hyperfiltration (17, 19, 25).

Studies have indicated that increased expression of the sodium-hydrogen exchanger isoform 1 (NHE-1) is linked to heart failure and may contribute to the development of hypertrophy and cardiac injury during ischemia and reperfusion (26, 27). SGLT2i have been shown to reduce myocardial fibrosis, a critical factor in heart failure progression. Additionally, they may promote the use of ketone bodies as an alternative energy source for the myocardium, potentially reducing the production of reactive oxygen species (ROS) (24, 26).

Of note, long-term use of SGLT2i may lead to a reduction in glycosuric efficacy, without a corresponding decline in cardiovascular benefits. This phenomenon is thought to result from compensatory mechanisms, such as increased SGLT1 activity and upregulation of SGLT2 expression. These findings support the rationale for dual SGLT1/SGLT2 inhibition as a potential therapeutic strategy (28–30).

### 3.2 Complications and side effects of SGLT2 inhibitors

The most common side effect of SGLT-2 inhibitors is polyuria, resulting from osmotic diuresis. Genital tract infections, affecting approximately 10-15% of women and less frequently in men, are another potential adverse effect (31, 32). Euglycemic diabetic ketoacidosis (euDKA), a rare but serious complication, has been primarily reported in patients with type 1 diabetes and may be precipitated by acute illnesses, inappropriate insulin dose reductions, or omissions (31).

Skin infections, such as Fournier’s gangrene, have been reported; their association with SGLT-2 inhibitors requires further confirmation through large, randomized trials (31). Genital fungal infections are up to four times more common in patients using SGLT-2 inhibitors (31, 32). The results of the CANVAS study showed a possible increased risk of amputations; however, neither this result nor the increased risk of fractures has been documented in other clinical trials of canagliflozin or other SGLT2 inhibitors, so further studies are required to establish a definitive link (32–35).

SGLT2 inhibitors should be discontinued in specific clinical scenarios to mitigate potential adverse events. Severe or recurrent genital mycotic infections and urinary tract infections warrant treatment suspension if they become problematic (36, 37). Patients presenting with euglycemic diabetic ketoacidosis require immediate cessation of SGLT2i therapy (36). Furthermore, it is recommended that SGLT2 inhibitors be discontinued during the perioperative period due to the risk of euDKA. This risk increases during the physiological stress associated with surgery and preoperative fasting, which can precipitate euDKA even in the absence of significant hyperglycemia. Additionally, treatment should be halted in elderly or frail individuals experiencing severe dehydration or orthostatic hypotension (36, 38).

Canagliflozin specifically should be discontinued in patients with a heightened risk of amputations, including those with a history of amputations, severe peripheral neuropathy, severe peripheral arterial disease, or active lower-limb ulcers or infections, as well as in individuals with an increased fracture risk, particularly those with previous osteoporotic fractures (34, 36, 37, 39). Although SGLT2 inhibitors do not typically increase the risk of AKI, their use should be halted during episodes of acute renal impairment (39). Although rare, cases of Fournier’s gangrene also necessitate immediate discontinuation of SGLT2 inhibitors to prevent further complications.

### 3.3 Mechanism of action of GLP1 receptor agonists

GLP-1 is a hormone belonging to the incretins that is produced in the gastrointestinal tract, by L cells, in response to the intake of nutrients, especially fat and glucose (40). Its release stimulates an increase in insulin secretion when stimulated by glucose at sufficient plasma levels (40). The effects of GLP-1 are not limited to the endocrinological component; there are receptors for these at the brain, liver, and gastrointestinal tract (41). It can influence renal function by increasing diuresis and natriuresis, and it can impact cardiac function by enhancing contractility and promoting cardiomyocyte survival. Additionally, GLP-1 can improve muscle insulin sensitivity and glucose uptake (42). These effects, including hemodynamic, metabolic, and anti-inflammatory actions, contribute to the cardio and renoprotective properties of GLP-1 RAs. By reducing intraglomerular pressure, decreasing inflammation, and mitigating oxidative stress, GLP-1 RAs can help preserve renal function, independent of glycemic control (43).

GLP-1 RAs like liraglutide and semaglutide, offer a therapeutic advantage over native GLP-1 by being resistant to degradation by dipeptidyl peptidase-4 (DPP-4). This resistance allows for prolonged exposure to GLP-1, resulting in sustained physiological effects. By activating GLP-1 receptors in the hypothalamus and brainstem, these analogs promote satiety, leading to reduced food intake and weight loss (40, 44).

Studies as Network Meta-analysis and clinical trials have shown that GLP1-RA can achieve a statistically significant decrease in HbA1C compared to placebo, as well as a weight reduction ranging between 1.3 and 8.65 kg (40, 45, 46). Furthermore, these analogues have the ability to stimulate natriuresis by inhibiting sodium reabsorption by decreasing the activity of sodium-hydrogen exchanger 3 (NHE3), resulting in a reduction in blood pressure in patients with diabetes (40, 46). In animal models, a modulatory effect has also been observed at the level of the carotid sinus, suggesting that this pharmacological class may influence sympathetic tone regulation during hyperglycemic states (47).

With regard to cardioprotection, several mechanisms have been proposed. GLP-1 RAs reduce macrophage adhesion to the endothelium, thereby inhibiting the formation of atherosclerotic plaques; they also suppress platelet activity, which may further contribute to cardiovascular protection (40). Moreover, these agents enhance cardiac glucose uptake and ATP production by increasing GLUT-1 translocation (40), and they modulate antioxidant, anti-inflammatory, and anti-apoptotic pathways (48).

### 3.4 Complications and side effects of GLP-1 RAs

Common side effects associated with GLP-1 receptor agonists (GLP-1 RAs) include gastrointestinal symptoms such as nausea (25–60%), diarrhea, and vomiting (5–15%) (16, 46). Although these adverse effects can be bothersome, they rarely lead to treatment discontinuation. Importantly, clinical trials have not demonstrated an increased risk of hypoglycemia with GLP-1 RAs compared to placebo (16). Less frequent side effects include injection site reactions, headaches, and nasopharyngitis (16).

While some studies have suggested a possible association between GLP-1 RAs and acute kidney injury (AKI), this has not been confirmed and appears to occur primarily in patients with underlying risk factors such as dehydration or severe gastrointestinal symptoms (49). Reports of preneoplastic pancreatic ductal disease exist, but experimental findings in mice have not been consistently replicated in human clinical trials. Although a potential association between GLP-1 RAs and gastrointestinal tumors has been proposed, current evidence does not support an increased risk of colorectal neoplasia (50).

GLP-1 RA therapy should be discontinued under specific clinical conditions to minimize risk. While a definitive causal relationship has not been established, the occurrence of acute pancreatitis necessitates immediate drug withdrawal, as recommended by the American Diabetes Association and the American College of Cardiology (37, 51). These agents are contraindicated in patients with a personal or family history of medullary thyroid carcinoma or multiple endocrine neoplasia type 2, based on findings from preclinical studies (21).

Treatment cessation should also be considered in patients who develop gallbladder complications, such as acute cholecystitis. Caution and close monitoring are warranted in individuals with preexisting diabetic retinopathy, due to a higher incidence of retinopathy-related complications observed in clinical trials, particularly with semaglutide (21, 37). In rare instances, severe hypersensitivity reactions, including anaphylaxis, require immediate discontinuation of GLP-1 RA therapy (52). Additionally, although uncommon, reported cases of diabetic ketoacidosis (DKA) associated with GLP-1 RAs also warrant prompt treatment cessation (53).

## 4 Cardiovascular results

### 4.1 Evidence of SGLT2 inhibitors in patients with DM2

**Figure 1:**
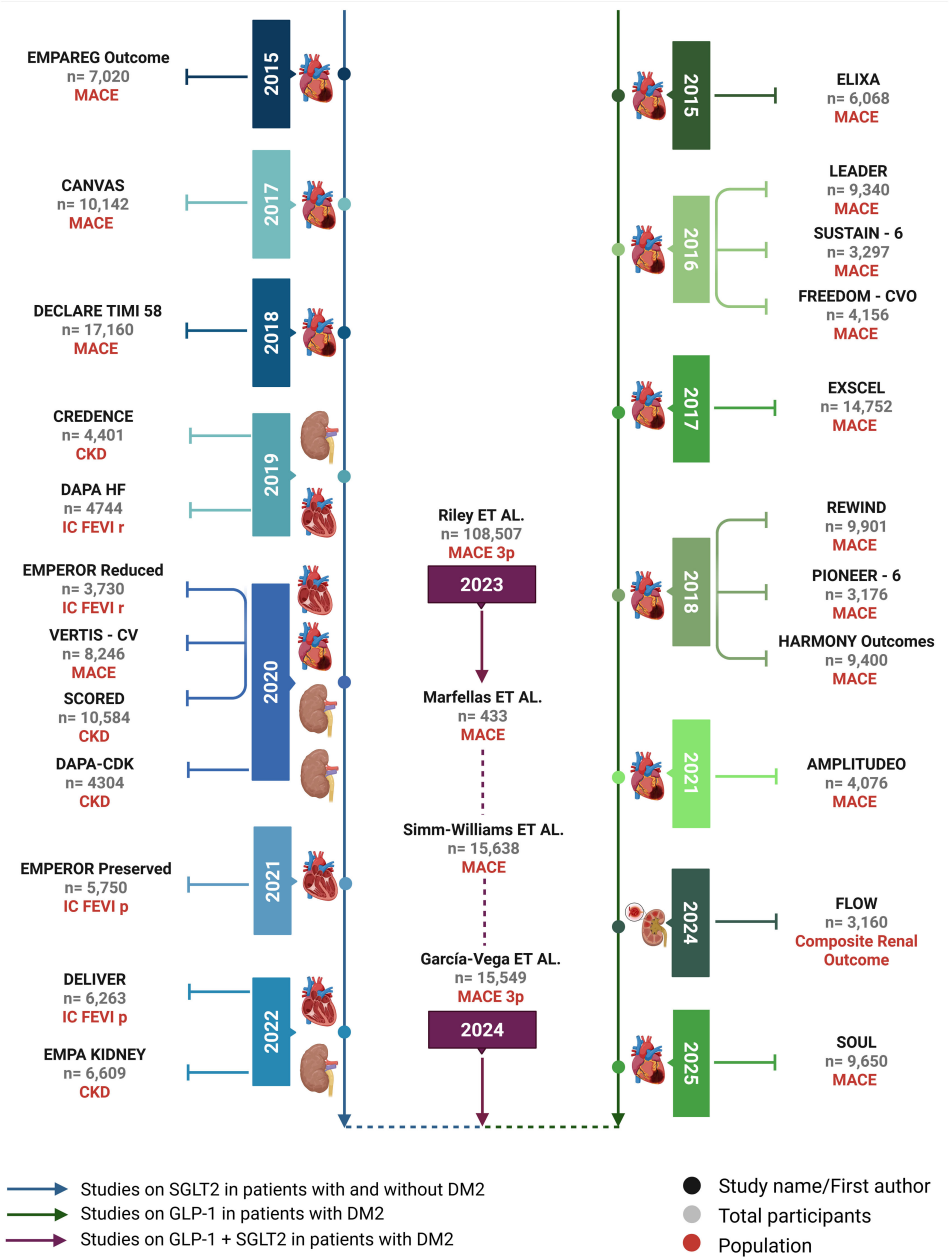
Timeline of major studies on SGLT-2i and GLP-1. The left axis displays key cardiovascular and renal outcome trials involving SGLT2 inhibitors, including studies in patients with and without T2DM. The right axis presents pivotal trials assessing GLP-1 receptor agonists in T2DM populations. The central column highlights observational studies and meta-analyses investigating the combined use of both drug classes. Study populations, primary outcomes (e.g., MACE, CKD, HF), and sample sizes are indicated for each trial.

The management of DM requires a long-term perspective, emphasizing the importance of medication safety over time. The Food and Drug Administration (FDA) mandates that clinical trials demonstrate the cardiovascular safety of anti-diabetic medications. To assess this, randomized trials typically evaluate the primary outcome of “major adverse cardiovascular events” (MACE), which includes cardiovascular death, non-fatal stroke, and non-fatal myocardial infarction (MI). This rigorous evaluation has led to the accelerated recognition of the cardioprotective, renoprotective, and vasoprotective effects of SGLT2i (54, 55) (**Figure 1**).

#### 4.1.1 Cardiovascular outcomes in SGLT2 inhibitors: safety studies

The EMPA-REG OUTCOME trial, which included 7020 patients with T2DM, was the first to demonstrate the cardiovascular protective effects of empagliflozin (54, 56). Patients were randomized to receive empagliflozin 10 mg, 25 mg, or placebo. After 3.1 years of follow-up, the trial showed a significant 14% reduction in the relative risk (RR) of MACE. Secondary outcomes included a 38% reduction in hospitalizations for HF and a 32% reduction in all-cause mortality (56).

Subsequent trials, such as CANVAS and DECLARE-TIMI 58, have further confirmed the cardiovascular benefits of SGLT-2 inhibitors. CANVAS demonstrated a 14% reduction in MACE in patients with T2DM and a high cardiovascular risk (34). CANVAS demonstrated a 14% reduction in MACE in patients with T2DM and a high cardiovascular risk. DECLARE-TIMI 58, which included a larger population with a lower baseline risk of CVD, did not show a significant reduction in MACE overall but did demonstrate a 27% reduction in hospitalizations for HF in a subgroup of patients with a history of myocardial infarction (54, 57). The explanation for the discrepancy in the reduction of MACE in patients treated with SGLT2i in DECLARE-TIMI 58 suggests that the benefits of SGLT2i may be more pronounced in patients with a higher risk of cardiovascular disease.

#### 4.1.2 Cardiovascular results in studies aimed at CVD

The CREDENCE clinical trial evaluated the effects of canagliflozin (100 mg daily) versus placebo in 4,401 patients with T2DM and CKD. While primarily designed to assess renal outcomes, the trial also demonstrated significant cardiovascular benefits. A 20% relative risk reduction in MACE, including myocardial infarction, stroke, and cardiovascular death and a 30% relative risk reduction in hospitalizations for HF (58).

The VERTIS trial, involving 8246 patients with T2DM, compared ertugliflozin 5 mg or 15 mg daily to placebo for 3.5 years. While ertugliflozin did not outperform placebo for MACE, it significantly reduced hospitalizations for HF by 30% (59).

The SCORED clinical study, which included 10,584 patients with T2DM and an estimated glomerular filtration rate (eGFR) of 25–60 ml/m²/1.73 m², evaluated the effects of sotagliflozin on cardiovascular death and hospitalization for HF. After 16 months, sotagliflozin demonstrated a significant reduction in the primary endpoint (HR: 0.74; 95% CI: 0.63 - 0.88; P<0.001) (60).

A meta-analysis of five double-blind placebo-controlled trials involving 46,969 patients showed that SGLT-2 inhibitors were associated with a 14% reduction in all-cause mortality and a 9% reduction in MACE. Hospitalizations for HF were also reduced by 31% compared to placebo. However, SGLT-2 inhibitors were associated with an increased risk of diabetic ketoacidosis (RR 2.59 CI95% 1.57, 4.27) and genital infections (RR 3.50 CI95% 3.09, 3.95) (17).

### 4.2 SGLT2i outcomes in patients with HF

The cardiovascular benefits of SGLT-2i are particularly evident in reducing the risk of hospitalization for HF, both in patients with and without T2DM. The DAPA-HF trial, which included 4744 patients with LVEF ≤40%, demonstrated a 26% reduction in the RR of hospitalization or emergency room visits for HF within 28 days of randomization (54, 61). The cardiovascular benefits of SGLT-2i are particularly evident in reducing the risk of hospitalization for HF, both in patients with and without T2DM. The DAPA-HF trial, which included 4744 patients with LVEF ≤40%, demonstrated a 26% reduction in the RR of hospitalization or emergency room visits for HF within 28 days of randomization. Furthermore, patients treated with dapagliflozin experienced a lower rate of cardiovascular death (9.6% vs. 11.5%), reflecting an 18% reduction in RR. In addition to these objective outcomes, the DAPA-HF study assessed quality of life using the Kansas City Cardiomyopathy Questionnaire (KCCQ) and found a significant improvement in the dapagliflozin-treated group compared to placebo, indicating a positive impact on patient-reported symptoms (62).

The EMPEROR-REDUCED trial, which included 3730 patients with HF and a left ventricular ejection fraction (LVEF) ≤40%, demonstrated a 25% reduction in the RR of cardiovascular death and HF hospitalization with empagliflozin 10 mg daily compared to placebo; hospitalization from IC was reduced approximately 30% (RR 0,70; IC del 95%, 0,58 a 0,85; P <0,001). While empagliflozin did not significantly reduce all-cause mortality, it improved renal outcomes, with a slower decline in GFR compared to placebo (63). Regarding renal outcomes, the decline in GFR was slower in the empagliflozin group, with an estimated –0.55 ml per minute per 1.73 m², compared with placebo, which was –2.28 ml per minute per year. This led to a reduction in the incidence of the composite renal outcome, defined as chronic dialysis, kidney transplantation, and a sustained decline in GFR (63).

Another trial that evaluated the efficacy and safety of empagliflozin was EMPEROR-Preserved; it included 5,988 patients with heart failure with preserved left ventricular ejection fraction, regardless of whether the patients had T2DM. Participants were randomly assigned to receive empagliflozin 10 mg or placebo once daily. There was a reduction in the RR in the primary outcome (cardiovascular death and hospitalization for heart failure) (RR 0.79, 95% CI 0.69-0.90). Highlighting the decrease in hospitalization for HF (0.71; 95% CI 0.60-0.83). In this study, it was unclear whether these benefits were preserved in subgroups of patients with higher left ventricular ejection fraction (>60%), as the result was not statistically significant (RR 0.87; 95% CI; 0.69-1.10) (64). It is also unclear whether this benefit was retained in patients who started treatment during the subacute phase, or in patients with an improved LVEF (54, 64).

In the DELIVER trial, in which participants were randomly assigned to receive dapagliflozin 10 mg or placebo once daily, 6263 patients with HF with LVEF >40% were enrolled. A significant reduction in RR was observed in the primary outcomes, such as cardiovascular death or worsening of HF, compared with placebo in the overall population (RR 0.82, 95% CI 0.73 to 0.92). Worsening of HF in the group of patients receiving dapagliflozin was 23% lower. These results were consistent both in patients with LVEF of 60% or more and in those <60%. In addition, the results were similar in prespecified subgroups, including patients with or without diabetes (65).

The SOLOIST-WHF trial evaluated the efficacy and safety of sotagliflozin in 1,222 patients with HF who were recently hospitalized for worsening of their baseline condition. This study demonstrated a significant risk reduction in a composite of cardiovascular death and worsening of HF (RR 0.67; 95% CI 0.52 to 0.85). Regarding hospitalizations and emergency department visits, the decrease in RR was 36%. These results were independent of LVEF (>50% or ≤50%) (66).

The DAPA-HF, EMPEROR-REDUCED, EMPEROR-Preserved, DELIVER and other trials mentioned above consistently demonstrate the efficacy of SGLT-2i in patients with HF, regardless of LVEF or the presence of diabetes. These studies establish SGLT-2i as a cornerstone therapy for HF across the disease spectrum (54, 61). Multiple meta-analyses performed on the most important studies have confirmed a significant risk reduction for a composite of cardiovascular death and hospitalization for heart failure, with the risk reduction for hospitalization being greater (67–69). In addition to the positive cardiovascular outcomes demonstrated in the studies discussed, early initiation of SGLT-2i is increasingly emphasized to reduce associated complications. This approach is supported by evidence on the results of studies such as the EMPULSE trial and the DICTATE-AHF trial (70–75).

SGLT2i have consistently demonstrated cardiovascular benefits in meta-analyses and recent studies, confirming their robust efficacy regardless of ejection fraction or the presence of diabetes. These findings support their role as a cornerstone therapy in the management of heart failure across the entire clinical spectrum (**Table 1**).

**Table 1:** Summary of the main studies with SGLT-2i.

Study/clinical trial	Drug (SGLT2)	Number of patients	Duration (years)	Primary outcomes	MACE reduction (%)	Hospitalization for HF reduction (%)	AlL-CAuse mortality (%)
EMPAREG	Empagliflozin	702	3.1	MACE, hospitalization for HF	14%	38%	32%
CANVAS	Canagliflozin	10,142	2.4	MACE, albuminuria reduction	14%	N/A	N/A
DECLARE-TIMI 58	Dapagliflozin	1,716	4.2	MACE, hospitalization for HF	Not significant	27%	N/A
CREDENCE	Canagliflozin	4,401	2.6	MACE, composite renal outcome	20%	N/A	N/A
VERTIS	Ertugliflozin	8,246	3.5	MACE, hospitalization for HF	Not significant	30%	N/A
SCORED	Sotagliflozin	10,584	1.5	CV death, hospitalization for HF	26%	N/A	N/A
DAPA-HF	Dapagliflozin	4,744	2.1	MACE, hospitalization for HF, CV death	26%	26%	18%
EMPA-KIDNEY	Empagliflozin	6,609	2	MACE, composite renal outcome	N/A	N/A	N/A
EMPEROR-REDUCED	Empagliflozin	3,730	1.9	Hospitalization for HF, CV death	25%	30%	N/A
EMPEROR-PRESERVED	Empagliflozin	2,997	2.2	Hospitalization for HF, CV death	21%	29%	N/A
DAPA CKD	Dapagliflozin	4,304	2.4	Decrease in eGFR, CV or Renal deaths	N/A	N/A	31%
DELIVER	Dapagliflozin	6,263	N/A	CV death, hospitalization for HF	23%	N/A	N/A
SOLOIST-WHF	Sotagliflozin	1,222	N/A	CV death, hospitalization for HF	36%	N/A	N/A

N/A, Not Available.

### 4.3 GLP-1 RAs in patients with DM2

Several randomized clinical trials have demonstrated the cardiovascular benefits of GLP-1 RAs in patients with T2DM. Five of these trials have shown superiority in reducing MACE compared to placebo, while all have confirmed the cardiovascular safety of GLP-1 RAs (76).

#### 4.3.1 Cardiovascular outcomes in GLP-1 RAs studies

The ELIXA trial, which included 6068 patients, was the first to investigate the cardiovascular effects of GLP-1 RAs. While lixisenatide 20 μg once daily did not demonstrate a significant reduction in the primary outcome of four-point MACE (RR1.02; 95% CI, 0.89–1.17), it was shown to be non-inferior to placebo in terms of cardiovascular safety (77).

The first study to demonstrate superiority over placebo was the LEADER study, in which 9,340 patients were assigned to either liraglutide or placebo. There was a significant reduction in the risk of MACE of 3 points (RR 0.87; 95% CI, 0.78-0.97). Regarding the cardiovascular mortality rate in patients with liraglutide, it was lower compared to placebo (RR 0.78; 95% CI, 0.66-0.93), and when analyzed in mortality due to any cause, it was also lower in the liraglutide group with a reduction of the RR by 15%. The rate of non-fatal stroke, non-fatal MI, and hospitalization for HF did not show significant differences (78). Semaglutide injection and CV impact was evaluated using the SUSTAIN-6 trial (designed as a non-inferiority trials), which primarily showed a reduction in the rate of non-fatal stroke events (RR 0.61; 95% CI, 0.38–0.99), as well as a decrease in the rate of MACE by 26% (79).

Other contemporary studies, such as the EXSCEL and FREEDOM trials evaluated the cardiovascular effects of exenatide, a GLP-1 RA (80, 81). EXSCEL found a 9% reduction in the relative risk (RR) of three-point MACE. While there were reductions in cardiovascular death (12%) and all-cause mortality (14%), the rates of fatal myocardial infarction (MI) and hospitalization for MI were not significantly different (80). The FREEDOM trial, with a median follow-up of 1.33 years, did not demonstrate a statistically significant difference in four-point MACE (RR 1.21; 95% CI, 0.90–1.63) (81).

The REWIND trial investigated the cardiovascular benefits of dulaglutide, a GLP-1 receptor agonist, in a population of 9,901 patients with T2DM aged 50+ and either established CVD or risk factors. The primary outcome was the first occurrence of a composite endpoint of non-fatal myocardial infarction, non-fatal stroke, or cardiovascular death. Dulaglutide significantly reduced the risk of this primary composite outcome by 12% compared to placebo (p=0.026). Gastrointestinal adverse events during follow-up were statistically significant (82).

Oral semaglutide has been studied in PIONEER 6 (designed as a non-inferiority trials), with a mean follow-up of 1.3 years and positive results for the primary endpoint: the MACE rate was reduced by 21% but did not achieve statistically significant differences. Furthermore, an important reduction in the number of deaths due to stroke was observed in the semaglutide treatment group (RR 0.49; 95% CI, 0.27-0.92). Regarding non-fatal myocardial infarction, there was no statistically significant difference (RR 1.18; 95% CI, 0.73-1.90) (83). For this drug, the ongoing SOUL trial has shown promising preliminary results, indicating a 14% reduction in the risk of MACE (84). The results of this study are expected in 2025.

The AMPLITUDE-O trial, which studied Efpeglenatide versus placebo with a mean follow-up of 1.81 years, demonstrated a significant 27% reduction in MACE. It also showed a reduction in heart failure (RR 0.61; 95% CI, 0.38–0.98) and cardiovascular mortality (RR 0.72; 95% CI, 0.50–1.03) (85).

Another study evaluating cardiovascular outcomes of GLP-1RAs was the HARMONY study. Albiglutide 30 mg was administered once weekly and enrolled a total of 9463 subjects aged ≥40 years with T2DM and established CVD. The albiglutide-treated group had a 3-point lower risk of MACE (0.78; 95% CI, 0.68–0.90). There was also a positive result for the rate of nonfatal myocardial infarction events, which was lower for the albiglutide group (RR 0.75; 0.61–0.90). However, the results were not positive for cardiovascular death (RR 0.93; 95% CI, 0.73–1.19) or stroke (RR 0.86; 95% CI, 0.66–1.14). It should be noted that this study did not evaluate hospitalization for HF (86).

A comprehensive meta-analysis by Giugliano et al. (2021) evaluated the cardiovascular benefits of GLP-1 RAs in patients with T2DM. The analysis, which included several of the above-mentioned, demonstrated a 14% reduction in the relative risk (RR) of MACE with GLP-1 RAs compared to placebo (87). Another meta-analysis also demonstrated that GLP-1s have a robust effect in reducing MACE (RR 0.87; 95% CI, 0.81-0.94) and death from any cause (RR 0.89; 95% CI, 0.83-0.95) (88). Positive results have also been found on HF, where GLP-1RAs could significantly reduce the incidence of hospital admission for heart failure by 11% (89).

Recent evidence has further substantiated the cardiovascular benefits of GLP-1 RAs. The SOUL trial (2025), a large-scale, double-blind, placebo-controlled study, evaluated once-daily oral semaglutide in 9,650 patients with T2DM and established atherosclerotic cardiovascular disease, chronic kidney disease, or both. Over a median follow-up of 49.5 months, oral semaglutide reduced the risk of MACE by 14% compared to placebo (HR 0.86; 95% CI, 0.77–0.96; p = 0.006), primarily driven by reductions in non-fatal MI and major adverse limb events. Approximately 27% of participants were on background SGLT2 inhibitor therapy at baseline; however, no significant interaction was observed, suggesting complementary cardiovascular protection mechanisms (90).

GLP-1 RAs significantly reduce the risk of MACE. Systematic reviews and meta-analyses confirm their overall cardiovascular benefits, demonstrating reductions in the relative risk of MACE, all-cause mortality, and hospitalizations for heart failure.

### 4.4 Role of GLP-1 RAs in arrhythmias and stroke

A meta-analysis by Liu et al. (2022) suggests that GLP-1 RAs may be associated with a reduced risk of atrial arrhythmias. This analysis included five trials with a total of 31,314 patients. While the study found that semaglutide specifically reduced the risk of atrial arrhythmias and atrial fibrillation (AF), other studies have reported conflicting results regarding the arrhythmia risk associated with GLP-1 RAs (91).

GLP-1RAs have been shown to significantly reduce postprandial levels of triglycerides, apolipoprotein (Apo) B48 and ApoC-III, independently of gastric emptying. In addition, liraglutide has been shown to significantly modify lipoprotein metabolism by reducing chylomicron production. Additionally, they exert a neuroprotective effect independently of blood glucose levels (92). Some of the antiatherosclerotic effects that contribute to stroke prevention are increased plaque stability, reduced vascular smooth muscle proliferation, and increased nitric oxide, all of which translate into improved endothelial function (92). This effect has been observed in several cardiovascular outcome clinical trials. GLP-1RAs have been shown to exert a protective factor by reducing stroke (RR 0.84; 95% CI, 0.77-0.93) (93) (**Table 2**).

**Table 2:** Summary of the main studies with GLP1-AR.

Study	Drug	Number of patients	Duration (years)	Primary outcomes	MACE reduction (%)	Cardiovascular mortality reduction (%)	Heart Failure hospitalization reduction (%)
ELIXA	Lixisenatide	6,068	N/A	No significant effect on MACE	Not significant	Not significant	Not evaluated
LEADER	Liraglutide	934	N/A	3-point MACE reduction	13%	22%	Not significant
SUSTAIN-6	Semaglutide	N/A	N/A	Reduction of non-fatal stroke, MACE	26%	N/A	Not evaluated
EXSCEL	Exenatide	N/A	3.2	3-point MACE risk reduction	9%	12%	Not significant
REWIND	Dulagluitde	9,901		3-point MACE risk reduction	12%	Not significant	Not significant
PIONEER 6	Semaglutide	N/A	1.3	Significant MACE reduction	21%	51%	Not evaluated
AMPLITUDE-O	Efpeglenatide	N/A	1.81	Reduction of MACE, Heart Failure	27%	28%	39%
HARMONY	Albiglutide	9,463	N/A	3-point MACE reduction	22%	Not significant	Not evaluated
SOUL	Oral Semaglutide	9650	4.1	MACE risk reduction	14%	Not significant	Not evaluated

N/A, Not Available.

### 4.5 Impact of SGLT-2 inhibitors and GLP-1 receptor agonists on kidney function

Multiple clinical trials have demonstrated the renoprotective effects of SGLT-2i and GLP-1 RAs in patients with T2DM and CKD. Studies such as EMPA-REG, CANVAS and DECLARE-TIMI 58, while not primarily focused on renal outcomes, have consistently shown that these medications can help preserve renal function (89, 94). In the EMPA-REG study, empagliflozin significantly reduced the risk for the composite renal outcome, which included doubling of serum creatinine, progression of macroalbuminuria, initiation of renal replacement therapy, or renal death (56). The CANVAS trial was a cardiovascular safety study; however, it suggested the presence of the renoprotective effects of canagliflozin in patients with T2DM and CKD. Canagliflozin-treated patients experienced a slower decline in GFR and an 18% reduction in the urea-albumin-creatinine ratio. Additionally, the risk of sustained doubling of serum creatinine, end-stage renal disease, and renal death was lower in the canagliflozin group (34).

Subsequent studies, such as CREDENCE, DAPA-CKD, and EMPA-KIDNEY, were specifically designed to investigate the renoprotective effects of SGLT-2i. The CREDENCE clinical trial evaluated the effects of canagliflozin 100 mg daily versus placebo in 4401 patients with T2DM and chronic kidney disease (CKD). With a mean follow-up of 2.6 years, the study demonstrated a 34% reduction in the relative risk (RR) of a composite renal outcome, including dialysis requirement, a decline in glomerular filtration rate (GFR) <15 ml/m²/1.73 m², kidney transplant requirement, doubling of creatinine, and cardiovascular or renal death (58).

The DAPA-CKD trial, which specifically evaluated the efficacy of dapagliflozin in patients with CKD, regardless of T2DM status, found a 39% reduction in the relative risk of a composite renal outcome, including a decline in GFR by at least 50%, end-stage renal disease, or renal or cardiovascular death. Dapagliflozin-treated patients had a lower incidence of these events 6.6% vs. 11.3% in the placebo group (0.61; 95% CI, 0.51 to 0.72) (95).

The results of the EMPA-KIDNEY study showed that its primary endpoint was the first occurrence of the composite outcome of kidney disease progression, defined as end-stage kidney disease, a sustained decline in eGFR to <10 mL per minute per 1.73 m², a sustained decline in eGFR of ≥40% from baseline, renal death, or cardiovascular death. The study included 6,609 patients, who were followed for 2.0 years. Emplagliflozin demonstrated a 28% reduction in the risk of the primary endpoint. In addition, it also showed a reduction in the risk of kidney disease progression by 29% (0.71; 95% CI; 0.62–0.81) (96).

Regarding GLP-1 RAs, the LEADER, SUSTAIN-6, REWIND, PIONEER 6 and AMPLITUDE-O studies have shown nephroprotective effect, especially in preventing the occurrence of macroalbuminuria. In the LEADER study, the group treated with liraglutide had a lower risk of nephropathy, with a reduction of 26% (78). In the SUSTAIN-6 trial, it was observed that semaglutide reduced the risk of nephropathy in patients treated with the drug compared to those treated with placebo (79). On the other hand, in the AMPLITUDE-O study, which included 4,076 patients, was shown that efpeglenatide significantly reduced the composite renal outcomes by 32% (a decrease in renal function or the occurrence of macroalbuminuria according to criteria defined in the study) (0.68; 95% CI, 0.57-0.79). This significant difference between the GLP-1 and placebo groups was mainly driven by a marked reduction in the incidence of macroalbuminuria (59). The REWIND trial also provided results consistent with previous studies. This study showed that the dulaglutide treated group had a lower risk of a composite renal outcome, (which includes the occurrence of macroalbuminuria, a 30% decrease in estimated glomerular filtration rate (eGFR) or the need for renal replacement therapy, (0.85, 95% CI 0 77–0.93), and the strongest statistically significant outcome was the reduction in the occurrence of macroalbuminuria (97).

The FLOW trial enrolled 3533 patients with T2DM and chronic kidney disease at high risk for kidney failure, cardiovascular events, and death. Participants were randomly assigned to receive subcutaneous semaglutide 1.0 mg weekly or placebo. The primary outcome was major kidney disease events. The semaglutide group experienced a 24% lower risk of the primary outcome compared to the placebo group (p<0.001). Additionally, the semaglutide group had an 18% lower risk of MACE (98). However, the SOUL trial, evaluating oral semaglutide, did not demonstrate a statistically significant reduction in major kidney disease events (90).

SGLT2 inhibitors have demonstrated significant renoprotective effects in patients with type 2 diabetes and CKD, reducing the risk of renal disease progression, the need for dialysis or kidney transplantation, and renal or cardiovascular mortality. GLP-1 RAs also confer renal benefits, particularly by reducing macroalbuminuria and slowing the progression of nephropathy. Together, these findings underscore the important role both drug classes play in renal protection for patients with type 2 diabetes.

### 4.6 Sex differences in cardiovascular outcomes

Subgroup analyses from the DAPA-HF trial revealed that women with heart failure experienced a reduction in the primary composite outcome with dapagliflozin (RR 0.79; 95% CI, 0.59–1.06), comparable to men (RR 0.73; 95% CI, 0.63–0.85), with no significant interaction by sex (62). Similar findings were reported in the CANVAS and EMPA-REG OUTCOME trials, where no significant sex-based differences were observed (34, 56). In contrast, a meta-analysis by Rivera et al. (2023) demonstrated a significant reduction in the primary composite outcome for both men (RR 0.77; 95% CI, 0.72–0.84; p < 0.00001) and women (RR 0.75; 95% CI, 0.67–0.84; p < 0.00001) receiving SGLT-2 inhibitors compared to placebo (99). Another meta-analysis evaluating three CVOTs with SGLT-2 inhibitors (N = 34,322) showed a reduction in MACE in men (RR 0.90; 95% CI, 0.83–0.97; p = 0.006), whereas in women, the risk reduction did not reach statistical significance (RR 0.88; 95% CI, 0.77–1.00; p = 0.06) (100).

Regarding safety, women experienced a higher incidence of genital infections while on SGLT-2 inhibitors, likely due to anatomical predisposition, which may impact treatment adherence (31).

For GLP-1 receptor agonists, the REWIND trial demonstrated a reduction in cardiovascular events with dulaglutide, with a trend toward greater benefit in women, although statistical significance was not achieved (RR 0.85; 95% CI, 0.71–1.02) vs. men (RR 0.90; 95% CI, 0.79–1.04; p = 0.60) (82). In the SUSTAIN-6 trial, no sex-based differences in cardiovascular outcomes were observed, indicating similar efficacy across genders (79). A meta-analysis including seven GLP-1 RA trials demonstrated significant MACE reduction in both men (RR 0.88; 95% CI, 0.82–0.93; p < 0.0001) and women (RR 0.88; 95% CI, 0.79–0.99; p = 0.03) (100).

In terms of safety, gastrointestinal adverse effects—particularly nausea—were common across both sexes, but women reported higher rates of intolerance, which may lead to treatment discontinuation (46). These observations underscore the importance of incorporating sex-specific factors when selecting GLP-1 RAs, favoring their use in women at elevated stroke risk, with potential dose adjustments to optimize adherence (8, 91).

The reduction in MACE with SGLT-2 inhibitors appears to be less consistent in women with type 2 diabetes than in men, while GLP-1 receptor agonists provide similar cardiovascular benefits across sexes.

### 4.7 Combined use of SGLT2i and GLP-1 RAs

Several studies have explored the combination of GLP-1 RAs and SGLT-2 inhibitors in patients with T2DM. Randomized clinical trials, such as DURATION-8, SUSTAIN-9, and AWARD-10, have documented the use of GLP-1 RAs in patients already receiving SGLT-2 inhibitors, demonstrating efficacy without adverse effects on the studied population (96, 97). Similarly, sub analyses of studies like EXSCEL, AMPLITUDE-O, HARMONY, CANVAS, DECLARE-TIMI 58, and VERTIS-CV have shown a positive impact with the concomitant use of GLP-1 RAs and SGLT-2 inhibitors, irrespective of whether the initial intervention involved GLP-1 RAs or SGLT-2 inhibitors (34, 57, 59, 80, 85, 101–105).

Among the studies evaluating GLP-1 RAs and SGLT-2 inhibitors, the study conducted by Riley et al. through the TriNetX network stands out. This study included approximately 2.2 million participants and compared cardiovascular outcomes among patients treated without GLP-1 RAs or SGLT-2 inhibitors, with one of these drug classes, or with a combination of both. After five years of follow-up in patients with T2DM, the GLP-1 RAs and SGLT-2 inhibitors groups demonstrated significant reductions in mortality, CHD, HF, AF, stroke, peripheral vascular disease, and CKD, regardless which one of the drugs or its combination were used. Notably, the simultaneous use of both pharmacological classes provided a significantly greater benefit in outcomes such as mortality, hospitalizations, heart failure, and CKD (106).

Building on the findings of the aforementioned trials, several meta-analyses have been conducted since 2019 to evaluate the safety and efficacy of combining GLP-1 RAs and SGLT-2 inhibitors. In 2019, Castellana et al. performed a meta-analysis focusing on patients with T2DM who required rescue medication for hyperglycemia and had a follow-up period of at least 24 weeks. This study demonstrated significant improvements in parameters such as HbA1c, body weight, and lipid profiles, along with a reduced need for rescue medications to control hyperglycemia (107), an effect that has been demonstrated again in other meta-analyses carried out subsequently (108). Additionally, to date it has been demonstrated to have an adequate safety profile by not increasing adverse effects with the combination of these pharmacological groups (108–111).

Among the prospective studies on this pharmacological combination, real-world evidence also plays a significant role. For instance, García-Vega et al. conducted a prospective study involving patients treated in Galicia between 2018 and 2022. The study included 15,549 patients who received either the combination therapy of GLP-1 RAs and SGLT-2 inhibitors or monotherapy with one of these drug classes. After an average follow-up of 19 months, the combination therapy did not demonstrate a reduction in coronary heart disease or ischemic stroke events compared to monotherapy. However, notable benefits were observed in other outcomes, including a 31% reduction in hospital admissions for heart failure and a 32% decrease in all-cause mortality (112).

Other high-profile studies on the use of GLP-1 RAs and SGLT-2 inhibitors combination therapy were conducted by Marfellas et al. (84, 96),. Marfellas et al. recruited patients with T2DM who had experienced acute myocardial infarction (AMI) and had been treated with one of these drug groups within the three months preceding the acute event. Patients with HbA1c >7% were initiated on the complementary drug to complete the GLP-1 RA + SGLT-2i combination therapy. The primary endpoint was a composite of cardiovascular death, recurrent acute coronary syndrome, and heart failure related to AMI after two years of follow-up. Among the 443 patients who completed the study, the combination therapy group demonstrated an ≈84% reduction in the primary endpoint (113).

Simms-Williams et al. conducted a population-based cohort study involving 15,638 patients receiving the combination therapy. Their findings revealed a ≈30% reduction in MACE compared to monotherapy. Regarding renal outcomes, the combination therapy showed a reduction in events that did not reach statistical significance when compared to SGLT-2i monotherapy. In contrast, when analyzing stroke outcomes, the effect was more pronounced with GLP-1 RAs, which provided the greatest contribution to the reduction of stroke events. However, a 57% reduction in renal events was observed compared to those receiving only GLP-1 RAs (114).

In 2024, Ahmad and Sabbour conducted a meta-analysis encompassing data from over 110,000 patients, encompassing 13 investigations that evaluated the combined use of GLP-1 RAs and SGLT-2 inhibitors. The findings demonstrated a significant reduction in all-cause mortality, with an odds ratio of 0.49 (95% CI [0.41–0.60]; p < 0.00001). Additional benefits included reductions in body mass index (BMI), blood pressure levels, HbA1c, and fasting blood glucose, observed after a minimum of six months of clinical follow-up (111).

Notably, the study by Marfellas et al. focused on a cohort of patients following a coronary event, providing insights into the cardiovascular benefits of the therapeutic combination in this high-risk population. In contrast, Simms-Williams et al. analyzed patients initially treated with GLP-1 RAs or SGLT2i, subsequently adding the second agent to evaluate the effects of combination therapy compared to monotherapy and differencing across groups (113, 114). The study by Riley et al., despite its retrospective design, represents the largest dataset to date, offering robust evidence on cardiovascular and renal outcomes associated with this therapeutic strategy (106). Lastly, the analysis by García-Vega et al. incorporated stroke outcomes, broadening the scope of evidence for this combination therapy in real-world settings (112). The meta-analyses reviewed highlight the favorable safety profile of the pharmacological therapy, alongside its efficacy in optimizing clinical parameters, particularly glycemic control and lipid profiles (**Table 3**).

**Table 3:** Summary of the main studies with combined use of GLP1-AR and SGLT2 inhibitors.

Study	Methodology	Number of patients	Duration (months)	Primary outcomes	Results	All-cause mortality (Reduction)	Heart failure consulting (Reduction)	CKD progression (Reduction)
Marfellas et al.	Prospective Cohort	443	24	Composite of the incidence of all-cause mortality, hospitalization for heart failure, acute coronary syndrome	Reduced incidence (≈84%) of cardiovascular events in patients with T2DM and AMI	N/A	N/A	N/A
Simms-Williams et al.	Population based cohort study	15,638	60	Cardio renal vascular disease with impatient consulting or mortality	GLP-1 RAs + SGLT-2 inhibitor combination was associated with a lower risk of MACE and serious renal events compared with either drug class alone.	29%	43%	57%
Riley et al.	Retrospective Cohort	108,507	60	All-cause mortality, hospitalization, Acute myocardial infarction	SGLT2i + GLP-1RAs combination therapy was associated with the greatest risk reduction in all-cause mortality.	75%	40%	28%
García-Vega et al.	Non-Concurrent prospective study	15,549	19	All-cause mortality, hospitalization or mortality by: coronary artery disease, heart failure, stroke	SGLT2i + GLP1ra reduces heart failure risk and all-cause mortality	32%	31%	Not evaluated
Castellana et al.	Meta Analysis	1,610	≈6	Efficacy and safety	The addition of GLP1 RAs to SGLT2i proved to be effective	Not evaluated	Not evaluated	Not evaluated
Ahmad and Sabbour	Meta Analysis of observational studies	≈110,000	3 to 60	Effectiveness and safety	Lower all-cause mortality and favorable improvements in cardiovascular, renal, and glycemic measurements.	51%	Not evaluated	Not evaluated

N/A, Not Available.

With respect to the SOUL trial, oral semaglutide demonstrated a 14% reduction in MACE outcomes, independent of concurrent SGLT2 inhibitor use. The combination therapy also exhibited a favorable safety profile in this trial (115).

To date, current evidence suggests that the combination of SGLT2 inhibitors and GLP-1 receptor agonists is generally well-tolerated. The adverse events observed align with the known safety profiles of each class used individually, with no indication of synergistic toxicity. This combination represents a promising therapeutic strategy for improving cardiovascular, renal, and metabolic outcomes in patients with T2DM.

## 5 Conclusions

The use of SGLT-2 inhibitors and GLP-1 RAs in patients with cardiovascular risk has been well-established over the past decade. When considering the initiation of these therapies, it is important to consider patient-specific factors. Patients with existing heart or kidney disease may benefit from starting SGLT-2 inhibitors therapy, while those with a higher risk of developing these conditions, such as obese patients and those with no cardiogenic stroke, may benefit more from GLP-1 RAs. However, it is essential to note that when additional pharmacological intervention is required to control diabetes mellitus, the choice between SGLT-2 inhibitors and GLP-1 RAs should be based on the patient’s individual needs and the specific medications already being used. The combination of SGLT-2 inhibitors and GLP-1 RAs offer additive benefits in reducing cardiovascular and renal risk in patients with diabetes and should be considered.
