# Correction: A decade of progress in type 2 diabetes and cardiovascular disease: advances in SGLT2 inhibitors and GLP-1 receptor agonists – a comprehensive review

**DOI:** 10.3389/fendo.2025.1663787

**Published:** 2025-08-20

**Authors:** David Aristizábal-Colorado, David Corredor-Rengifo, Santiago Sierra-Castillo, Carolina López-Corredor, David-Alexander Vernaza-Trujillo, Danilo Weir-Restrepo, Juan S. Izquierdo-Condoy, Esteban Ortiz-Prado, Jorge Rico-Fontalvo, Juan-Esteban Gómez-Mesa, Alin Abreu-Lomba, Wilfredo-Antonio Rivera-Martínez

**Affiliations:** ^1^ Internal Medicine Department, Universidad Libre, Cali, Colombia; ^2^ Grupo Interinstitucional de Medicina Interna (GIMI1), Universidad Libre, Cali, Colombia; ^3^ Epidemiology Department, Universidad Centros de Estudios en Salud (CES), Medellín, Colombia; ^4^ Medicine Program, Universidad Santiago de Cali, Cali, Colombia; ^5^ Department of Public Health, Pontificia Universidad Javeriana, Cali, Colombia; ^6^ Clinical Research Center, Clínica Imbanaco, Cali, Colombia; ^7^ Internal Medicine Department, Universidad Centros de Estudios en Salud (CES), Medellin, Colombia; ^8^ One Health Research Group, Universidad de las Américas, Quito, Ecuador; ^9^ Department of Nephrology, Faculty of Medicine, Universidad Simón Bolívar, Barranquilla, Colombia; ^10^ Latin American Society of Nephrology and Arterial Hypertension (SLANH), Ciudad de Panama, Panama; ^11^ Cardiology Department, Fundación Valle del Lili, Cali, Colombia; ^12^ Department of Health Sciences, Universidad Icesi, Cali, Colombia; ^13^ Endocrinology Department, Clínica Imbanaco, Cali, Colombia; ^14^ Endocrinology Department, Universidad de Antioquia, Medellín, Colombia

**Keywords:** cardiovascular outcomes, SGLT2 inhibitors, GLP-1 agonists, combination therapy, heart failure, renal outcomes

The affiliations “Department of Public Health, Pontificia Universidad Javeriana, Cali, Colombia” and “Clinical Research Center, Clínica Imbanaco, Cali, Colombia” were omitted for author David Alexander Vernaza Trujillo.

The original version of this article has been updated.

